# Feature-Based Attention in Early Vision for the Modulation of Figure–Ground Segregation

**DOI:** 10.3389/fpsyg.2013.00123

**Published:** 2013-03-18

**Authors:** Nobuhiko Wagatsuma, Megumi Oki, Ko Sakai

**Affiliations:** ^1^The Zanvyl-Krieger Mind/Brain Institute, Johns Hopkins UniversityBaltimore, MD, USA; ^2^Riken Brain Science InstituteWako, Japan; ^3^Department of Computer Science, University of TsukubaTsukuba, Japan

**Keywords:** feature-based attention, figure–ground segregation, border-ownership, early vision, psychophysical experiment, computational model

## Abstract

We investigated psychophysically whether feature-based attention modulates the perception of figure–ground (F–G) segregation and, based on the results, we investigated computationally the neural mechanisms underlying attention modulation. In the psychophysical experiments, the attention of participants was drawn to a specific motion direction and they were then asked to judge the side of figure in an ambiguous figure with surfaces consisting of distinct motion directions. The results of these experiments showed that the surface consisting of the attended direction of motion was more frequently observed as figure, with a degree comparable to that of spatial attention (Wagatsuma et al., 2008). These experiments also showed that perception was dependent on the distribution of feature contrast, specifically the motion direction differences. These results led us to hypothesize that feature-based attention functions in a framework similar to that of spatial attention. We proposed a V1–V2 model in which feature-based attention modulates the contrast of low-level feature in V1, and this modulation of contrast changes directly the surround modulation of border-ownership-selective cells in V2; thus, perception of F–G is biased. The model exhibited good agreement with human perception in the magnitude of attention modulation and its invariance among stimuli. These results indicate that early-level features that are modified by feature-based attention alter subsequent processing along afferent pathway, and that such modification could even change the perception of object.

## Introduction

Among the numerous objects that are projected onto the retina, attention selects objects that come into our perception (Posner, 1980; Itti and Koch, 2001). Such selection can be made based on space, feature, and object. In all cases, attention modulates the activity of neurons in the visual cortex (McAdams and Maunsell, 1999; Treue and Maunsell, 1999; Martinez-Trujillo and Treue, 2004; Gregoriou et al., 2009; Cohen and Maunsell, 2011; Wagatsuma et al., 2011), thus enhancing perception or even changing the perception (e.g., Palmer et al., 1993; Solmon et al., 1997; Hasson et al., 2001; Carrasco et al., 2004; Mitchell et al., 2004). For example, Tzvetanov et al. (2006) have shown psychophysically that motion direction acts as a feature and motion-discrimination is enhanced around an attended motion direction. Physiological studies have reported that a majority of cells in V2 are selective to border-ownership (BO; Zhou et al., 2000), which is a precursor of figure–ground (F–G) segregation, and that spatial attention modulates the activities of the cells (e.g., Qiu et al., 2007).

Although the neural mechanisms underlying the modulation of perception by attention have been studied extensively (e.g., Deco and Lee, 2004; Liu et al., 2007a; Ling et al., 2009; Reynolds and Heeger, 2009; Baluch and Itti, 2011), the manner *via* which the top-down signal mediating attention affects the bottom-up flow of F–G segregation has not been clarified. Top-down attention appears to modulate lower-level features, with modulation of contrasts in V1 at the lowest level (Lee et al., 1999; Paradiso, 2002; Carrasco, 2011). The afferent transmission beginning in the low-level features should gradually establish the perception as the signal progresses through the hierarchy of the visual pathway. Our previous computational study suggested that the luminance contrast in V1 within an attended location is modified by spatial attention, so that further processing in V2 is altered, to modulate the perception of F–G (Wagatsuma et al., 2008). Specifically, spatial attention strengthens the low-level feature contrast extracted in V1, followed by the feeding of this modified contrast to BO-selective cells in V2 *via* the surround modulation. As BO is determined based on the balance of surround low-level feature contrast between the sides with respect to its Classical Receptive Field (CRF) (Sakai and Nishimura, 2006), the response of a BO-selective cell is enhanced if spatial attention is directed to its preferred direction. Our model of spatial attention accounted for the mechanism of F–G switching and reproduced the human perception. It is natural to expect that a neural mechanism similar to this spatial attention underlies feature-based and object-based attention, as a common mechanism for which attention affects bottom-up flow. Recent studies support this expectation. A physiological study has implied that spatial- and feature-based attention affect local populations of cells in similar ways (Cohen and Maunsell, 2011). A large-scale simulation study has suggested that the differences between spatial and feature-based attention emerge from differential top-down influences on visual cortical networks rather than from the presence of different neural circuits specialized for the two types of attention (Wagatsuma et al., 2012).

**Figure 1:**
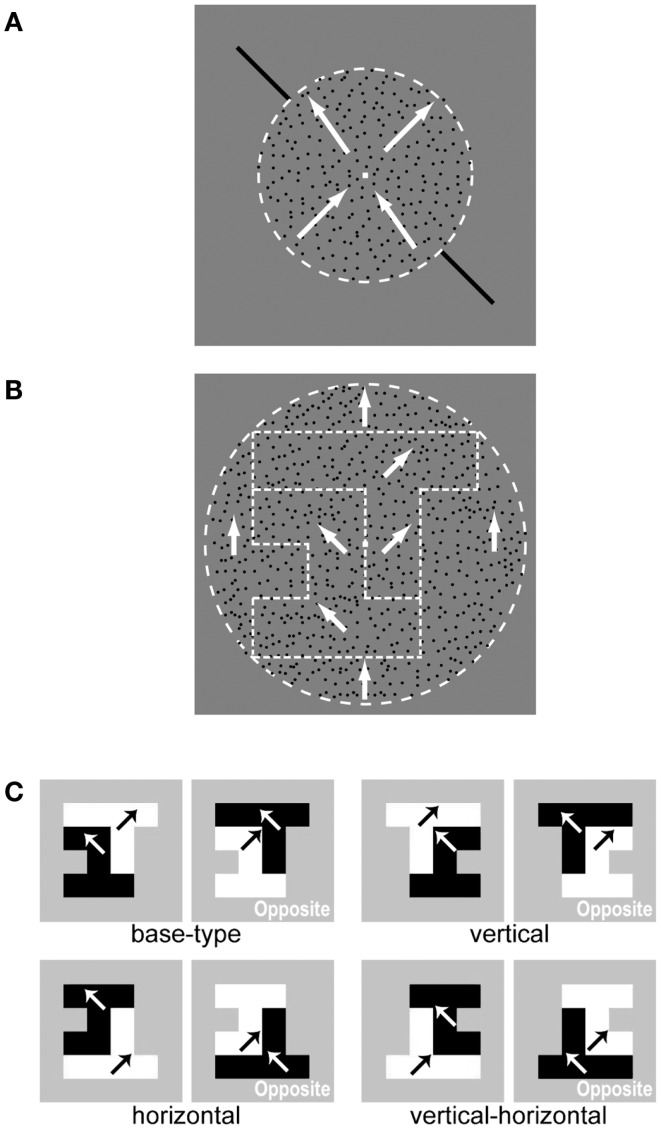
**Stimuli used in the psychophysical experiments**. **(A)** An illustration of stimulus settings for the motion-discrimination task aimed at directing attention to a specific motion direction. The two superimposed surfaces of the RDP moving toward orthogonal directions (shown by white arrows) are presented within a circular region (indicated by the white dashed line). These two circular surfaces are transparently overlapping in space. The two black bars (reference bars) drawn outside the circular region provided the reference for motion directions. Participants were asked to judge the motion direction of an RDP that moved toward (but not exactly to) one of the reference bars. Specifically, the participants were instructed to answer whether the RDP moved clockwise or counterclockwise with respect to the reference bar. **(B)** An illustration of stimulus settings for the DOF-discrimination task. The stimulus consisted of two random-block objects in which the DOF was ambiguous at the boundary. The two objects were segregated from each other and from the circular background by difference in motion direction. The white arrows indicate the motion direction of the RDP within each region (boundaries are indicated by white dashed lines). Participants were instructed to report on which side of the fixation aid (a small square at the center) appeared in front (figure). **(C)** Mirror images of an example stimulus with respect to the vertical midline, horizontal midline, and both. Black and white arrows indicate the motion direction of the RDP within each object. A set of stimuli with a polarity that was opposite to that of motion directions (right panel) was also presented. We used these mirror images and two motion polarities to cancel biases in the perception of DOF.

We investigated whether and how feature-based attention modulates F–G segregation. First, we conducted psychophysical experiments to examine whether BO perception is modified by feature-based attention. In these experiments, the attention of participants was drawn to a specific motion direction *via* a motion-discrimination task similar to Tzvetanov’s et al. (2006) experiments (Figure 1), and participants were then asked to judge the side of figure in an ambiguous figure with surfaces consisting of distinct motion directions. The results of these experiments showed that feature-based attention alters perception, leading to the observation of the figure on the surface consisting of the attended direction of motion, with a degree comparable to that of spatial attention. Furthermore, these experiments showed that perception is dependent on the distribution of the motion direction difference along the border between figure and ground, and independent of the object size. Second, we examined computationally whether a neural mechanism similar to that of spatial attention underlies the perception. Specifically, based on the results of the psychophysical experiment, we proposed that feature-based attention modulates the feature contrast or edge, specifically motion direction differences, in early vision and that this modulation of the low-level feature contrast changes the activities of BO-selective cells directly *via* the surround modulation, thus modifying the perception of BO. We constructed a V1–V2 model and performed simulations of the model with ambiguous figures mimicking our psychophysical experiments. The model exhibited good agreement with human perception regarding the magnitude of attention modulation and reproduced its invariance among stimuli. These results suggest that early-level features that are modified by feature-based attention alter subsequent processing along the afferent pathway, and that such modification could even change the selection of an object during F–G segregation.

## Materials and Methods

### Psychophysical experiments

#### Stimuli

We used two types of stimuli consisting of a moving Random Dots Patterns (RDP): a cue stimulus for drawing attention to a specific motion direction (Figure 1A) and a test stimulus for examining the perception of Direction Of Figure (DOF, Sajda and Finkel, 1995) (Figure 1B). The cue stimulus aimed to lead the feature-based attention of participants to a specific motion direction (Figure 1A). Two circular surfaces of moving RDPs were transparently superimposed in space within a diameter of 8° (degree of the visual angle). One surface included an RDP moving toward the right and upward (35°, 45°, or 55° clockwise from the vertical) and the other surface included an RDP moving toward the left and upward (−35°, −45°, or −55°). We superimposed two surface with distinct directions of motion to effectively yield motion-based attention (Tzvetanov et al., 2006). All RDPs had a luminance of 3.84 cd/m^2^ and a speed of 4°/s (for Experiments 1 and 2) or 5°/s (for Experiment 3). We adopted faster speed for Experiment 3 in order to cancel out stimulus complexity that may lower the effect of attention. Diagonal bars (11° × 11°, 3.84 cd/m^2^) oriented either right and upward (45°) or left and upward (−45°) were chosen and drawn outside the circles, as references. Participants were instructed to fixate a small square (0.2° × 0.2°, 162.4 cd/m^2^) located at the center of the screen and judge whether the motion direction of RDP was more “counterclockwise” or “clockwise” with respect to the reference bars that were presented at the time (motion-discrimination task). This method for drawing attention to a specific direction of motion has been utilized by Tzvetanov et al. (2006). They showed that spatial interactions among sensory information were eliminated as far as attention was directed to a target direction of motion.

The DOF of a test stimulus was designed to be ambiguous along the vertical through a fixation point (Figure 1B). Ambiguous figures were generated from the combinations of pseudo white-noise random-block objects (see the Appendix for the generation algorithm; Sakai and Nishimura, 2006; Wagatsuma et al., 2008; Sakai et al., 2012). The shape of each object was generated from up to six square blocks that were placed within a 4 × 4 grid, for Experiments 1 and 2, or eight square blocks placed within a 6 × 6 grid, for Experiment 3. The RDP within one object moved toward +45° and the other toward −45°, surrounded by a RDP moving upward with a diameter of 12°. Differences in motion direction at borders evoked the segregation of regions and generated two surfaces in the shape of random-block objects against the background. The luminance and speed of RDPs were identical to those used for the cue stimulus. Participants were instructed to judge which side, the left or right, appeared in front of the other with respect to the fixation point, i.e., to report the DOF at the fixation point (DOF-discrimination task). To cancel any biases in the perception of DOF, we utilized mirror images with respect to the vertical and horizontal midlines and images with a polarity that was opposite to that of motion directions (Figure 1C; Wagatsuma et al., 2008). We prepared 40 test stimuli in total (five types of ambiguous figures × four mirror images × two motion polarities). The combination of four types of cue stimuli yielded 160 trials, which was considered as one set in this experiment. We performed two sets for each subject.

#### Experimental procedure

**Figure 2:**
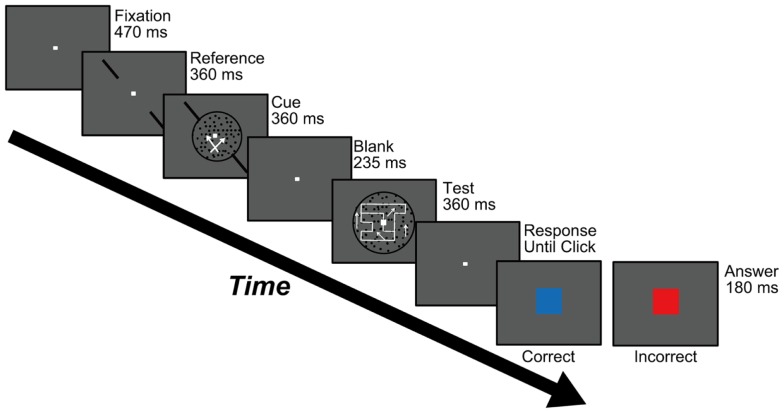
**Procedure used in Experiments 1 and 2**. Participants were asked to carry out a motion-discrimination task (the stimulus is shown during the “Cue” screen) and a DOF-discrimination task (during the “Test” screen). Participants were instructed to respond to both tasks at the end of each trial using a two-alternative forced choice (2AFC) paradigm. See text for details.

Figure 2 shows the procedure used to perform Experiments 1 and 2, which aimed to examine feature-based attention in the perception of DOF. The experiments started with the presentation of a fixation aid (0.2° × 0.2°) at the center of the screen for 470 ms. Participants were instructed to gaze at the fixation aid during the experiment. A pair of reference bars for motion-discrimination was presented along either the left/upward or right/upward diagonals for 360 ms. A cue stimulus appeared within a circle of 8° for 360 ms with the simultaneous presentation of the diagonal reference bars (Figure 1A). Participants were instructed to judge the direction of dot motion with respect to the reference bars (clockwise or counterclockwise) while ignoring the RDP moving orthogonally to the reference bars, and to report this judgment at the end of the trial. This task aimed to direct attention to a specific motion direction. Subsequently, a blank screen was presented for 235 ms and a test stimulus for DOF-discrimination (Figure 1B) was presented inside a circle of 12° for 360 ms. Participants were asked to report which of the left or right object was perceived as figure in the test stimulus by clicking mouse buttons sequentially. At the end of each trial, participants were told whether the response regarding motion-discrimination was correct or not; a blue square (5.7° × 5.7°) was displayed for 180 ms for a correct response and a red square was displayed for 180 ms for an incorrect response. This feedback aimed to enhance the attention of individuals to a specific motion direction. The order of presentation of each condition (the combination of the orientation of reference bars and the directions of moving dots in cue and test stimuli) was randomized. This method allowed us to examine the perception of DOF while attending to a specific direction of motion. This procedure was similar to our previous psychophysical experiments for testing whether the behaviors of the model for spatial attention agreed with human perception of attention modulation in BO determination (Wagatsuma et al., 2008).

#### Observers and apparatus

Six male and two female participants in their twenties with normal or corrected-normal vision participated in the experiments. They were familiar with visual psychophysics but not aware of the aim of the experiments. The experiment was conducted using a 21′′ CRT monitor (GDM8411; Silicon Graphics Inc.) at a refresh rate of 85 Hz, controlled by a PC (Precision 360-n; Dell). The monitor was placed at a distance of 57 cm in front of the participants in Experiments 1 and 2, and at 85.5 cm in Experiment 3. Shapes of random-block objects in Experiment 3 were more complex than those in Experiments 1 and 2 (see Difference in Motion Direction for the Attention Modulation of DOF – Experiment 3 and the Appendix for details) thus we drew them slightly larger to assure similar resolution on the monitor. For presenting their entire shapes within the same visual angle as in Experiments 1 and 2, the distance between the monitor and participants for Experiment 3 were set slightly longer than Experiments 1 and 2. Note that we did not compare directly the degree of attention modulation among the Experiments.

### The model

#### Model architecture

**Figure 3:**
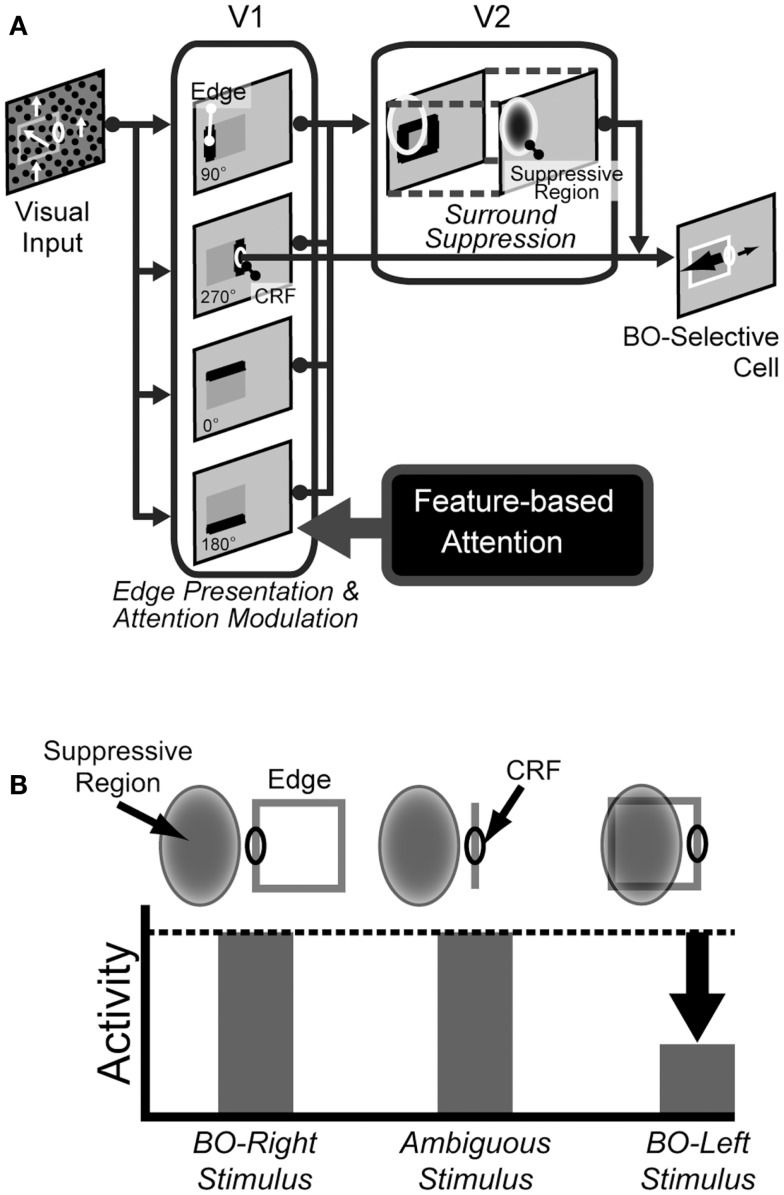
**The proposed model**. **(A)** An illustration of the model architecture. The abstract model consists of V1 and V2 stages, together with the source of top-down feature-based attention, presumably MT. The activities of BO-selective cells in V2 stage are determined by the low-level feature contrasts or edges, specifically the motion direction differences, from V1 stage. Top-down, feature-based attention operates on V1 only and enhances feature contrast or edge at object boundaries. **(B)** An illustration of the mechanism for a BO-Right selective model cells (Sakai and Nishimura, 2006; Sakai et al., 2012). This example cell has suppressive region (shaded ellipse) on the left of the CRF. If a figure (square) falls onto the left side, the edge within the surrounding suppressive region inhibits the activity of the cell. Therefore, the activity of the cell is stronger if a figure is placed on the right of the CRF, indicating BO-right selectivity.

The computational study (Sakai and Nishimura, 2006) proposed that the cortical mechanism underlying BO coding and F–G segregation involved the surrounding contrasts and surround modulation observed in early visual areas (Jones et al., 2001, 2002). Although this model was rather abstract model in the sense that BO was determined solely from contrast balance without biophysical details, the model not only reproduced the characteristics of BO-selective cells but also was supported by psychophysical experiments (Sugihara et al., 2007; Sakai et al., 2012). Based on this BO signaling mechanism depending on surrounding contrasts and edges, we proposed the model of spatial attention for BO modulation (Wagatsuma et al., 2008). In our previous model, spatial attention modulates luminance contrast in V1, which then alters the activities of BO-selective cells in V2. This spatial attention model explained the mechanism of the switch of F–G and reproduced qualitatively and quantitatively the human perception of DOF. It is expected that a common framework to this spatial attention model underlies the mechanism of different types of attention. Our proposed model for feature-based attention shared the framework used for spatial attention. We developed the present model to investigate the role of feature-based attention in the modulation of DOF that was observed in our psychophysical experiments. The proposed model was composed of two stages: V1 and V2, as illustrated in Figure 3A. In this proposed abstract model, top-down feature-based attention from higher visual areas, presumably MT, was directed to a specific feature such as the motion direction, which led to selective enhancement of the low-level feature contrasts or edges presented in V1 stage. This alternation resulted in the modulation of model BO-selective cells in V2 stage, because the responses of the cells were based on the surrounding modulation by the low-level feature contrast extracted in early vision (Figure 3B; Jones et al., 2001; Jones et al., 2002; Ozeki et al., 2009; Sakai and Nishimura, 2006; Sugihara et al., 2007; Sakai et al., 2012). Here, we consider local, component feature that appeared to be processed in V1, rather than complex pattern presentation apparent in MT. In the case of our experimental stimuli, edges or feature contrast are formed by the differences in motion directions. Note that there is no direct attention effect for the BO determination in V2 stage.

#### V1 stage

We consider local, component feature contrast that appeared to be processed in V1, rather than patterns apparent in MT. In our model, the low-level feature contrast in the V1 stage played a key role for the determination of the DOF, i.e., the responses of the V2 stage was independent of the distribution of the features itself. The V1 stage modeled the primary visual cortex, in which local edges or low-level feature contrasts, specifically the motion direction differences, are presented and are modulated by the top-down feature-based attention. The input to the V1 stage is provided by a stimulus map composed of edge detector *E*_θ_(*x*, *y*), akin to the topographic representation in V1 cells (Craft et al., 2007; Mihalas et al., 2011). Indices *x* and *y* are spatial positions. Orientations, θ, were selected among 0°, 90°, 180°, and 270°. Input to the V1 stage, *E*_θ_, had a resolution of 112 × 112 pixels. The activities of *E*_θ_ depended on the magnitude of the local contrast of the feature. In the case of our psychophysical stimuli, the activities of *E*_θ_ on the border between two objects are higher than that between objects and backgrounds.

Top-down feature-based attention was applied to the V1 stage and modulated the feature contrast or edge, specifically the motion direction differences, *E*_θ_(*x*, *y*). The modulated edges, $I_{\theta }^{V1}$, are given by the following equation, as proposed by Lee et al. (Lee et al., 1999; Peters et al., 2005):

(1) $$\begin{array}{llll} & I_{\theta }^{V1}\left(x,y\right)= & & \\ & \frac{E_{\theta }{{\left(x,y\right)}^{\gamma }{}^{I}}^{{}^{\text{Attn}}}}{{S^{\delta }{}^{I}}^{{}^{\text{Attn}}}+\underset{\theta }{\sum }{\left(\frac{1}{\left(2M+1\right)\left(2N+1\right)}\overset{N}{\underset{n=-N}{\sum }}\overset{M}{\underset{m=-M}{\sum }}E_{\theta }\left(x+n,y+m\right)\right)}^{\delta I^{\text{Attn}}}} \end{array}$$

where *I*^Attn^ implies whether top-down feature-based attention is directed to this feature and represents the magnitude of attention. If *E*_θ_(*x*, *y*) were the edges formed by motion direction differences with respect to the attended direction of motion, we used *I*^Attn^ = 1.0. In the case of the attention to the opposite feature, we set *I*^Attn^ = 0.0. γ and δ are constants. *S* is a semisaturation constant that prevents the denominator to be zero. The constant *S* is relatively sensitive to the simulation results. However, this semisaturation constant ranging between 2.0 and 3.5 showed marked attention modulation of model BO-selective cells. In our simulations, we used γ = 4.0, δ = 3.0, and *S* = 3.05. If the ratio of γ to δ is fixed, simulation results are almost the same even when these two constants are doubled. These constants were determined based on our previous model (Wagatsuma et al., 2008) and were fixed throughout all simulations. Equation 1 indicates that top-down feature-based attention in V1 stage enhances the edges such as the differences in the motion direction with respect to the specific feature.

#### V2 stage

A mathematical description of the surround suppression of a model BO-selective cell in V2 stage is given here. The activity of the model BO-selective cells is determined from the edge signals formed by motion direction differences transmitted from V1 stage (Figure 3B; Sakai and Nishimura, 2006; Sakai et al., 2012).

First, V2 stage pools the edge signals that are transmitted from V1 stage and have been modulated by top-down feature-based attention over space and orientation:

(2) $$O^{1}\left(x,y\right)=I_{0}^{V1}\left(x,y\right)+I_{90}^{V1}\left(x,y\right)+I_{180}^{V1}\left(x,y\right)+I_{270}^{V1}\left(x,y\right)$$

where *O*^1^ represents the pooling of the modulated edge signals from V1 stage.

Second, the surrounding signal, $O_{N}^{2}$, is given by a linear combination of edge signals from suppressive regions, which are defined by Gaussian functions, as illustrated in Figure 3B:

(3) $$\begin{array}{llll} & O_{N}^{2}\left(x,y\right)= & & \\ & c\overset{k_{x}}{\underset{i=1}{\sum }}\overset{k_{y}}{\underset{j=1}{\sum }}\left(R_{N}\left(i,j\right)O^{1}\left(x-\frac{k_{x}}{2}+i\right)\left(y-\frac{k_{y}}{2}+j\right)\right) \end{array}$$

where the index *N* represents the type of model BO-selective cells, which are distinguished by the size and the location of their surrounding suppressive regions; *R_N_* represents the suppressive regions of the model BO-selective cells. Physiological studies have reported a diversity of characteristics of BO-selective cells in V2 (Zhou et al., 2000; Qiu et al., 2007). The size and location of these suppressive regions determine the properties of and reproduce various BO selectivity. We implemented 10 types of suppressive regions from a pool of Gaussians generated randomly (Sakai and Nishimura, 2006; Sakai et al., 2012). These are common to a previous spatial attention work (Wagatsuma et al., 2008); *k_x_* and *k_y_* indicate the spatial extent of suppressive regions; and *c* is the connection strength.

Third, the response of model BO-selective cells, $I_{N}^{V2}$, was computed from the linear summation of the CRF signal, *O^1^*, and the surround signal, $O_{N}^{2}$.

If $O^{1}\left(x,y\right)-O_{N}^{2}\left(x,y\right)>0$,

(4) $$I_{N}^{V2}\left(x,y\right)=O^{1}\left(x,y\right)\times \left(O^{1}\left(x,y\right)-O_{N}^{2}\left(x,y\right)\right)$$

otherwise

(5) $$I_{N}^{V2}\left(x,y\right)=0$$

For the determination of DOF, the activities of model BO-selective cells were pooled, for representing the population activities. For the sake of simplicity, we took the summation of all activities of BO-selective cells that prefer right side and those prefer left side, respectively. Based on the magnitude of the two values, the dominant population was considered to own the border. Note that feature-based attention did not act directly on the model BO-selective cells in V2 stage.

## Results

### Feature-based attention in the perception of DOF – Experiment 1

**Figure 4:**
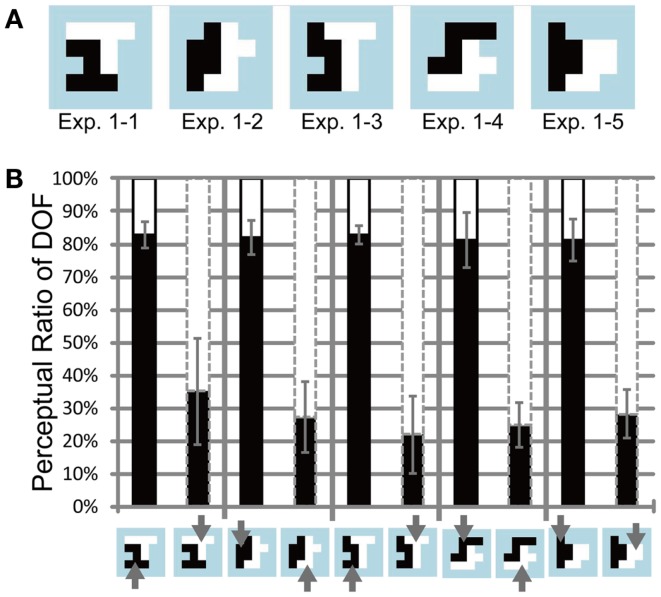
**Test stimuli and results of Experiment 1**. **(A)** Five base-type random-block stimuli with an ambiguous DOF at the center. The black and white regions indicate the distinct motion direction of the RDP. The areas of the black and white regions are equal. **(B)** Results of Experiment 1, using four participants. The black bars indicate the apparent perception of DOF toward the left with respect to the center for each base-type stimulus (bottom panel). The white bars (above the black bars) represent the perception of DOF toward the right. The black solid and gray dashed contours around the bars indicate that black and white objects, respectively, were attended. Arrows on the bottom panel also present the attention conditions. The regions with attended motion direction were perceived more frequently as figure than were the alternative regions. Error bars represent the standard error. See main text for details.

**Figure A1:**
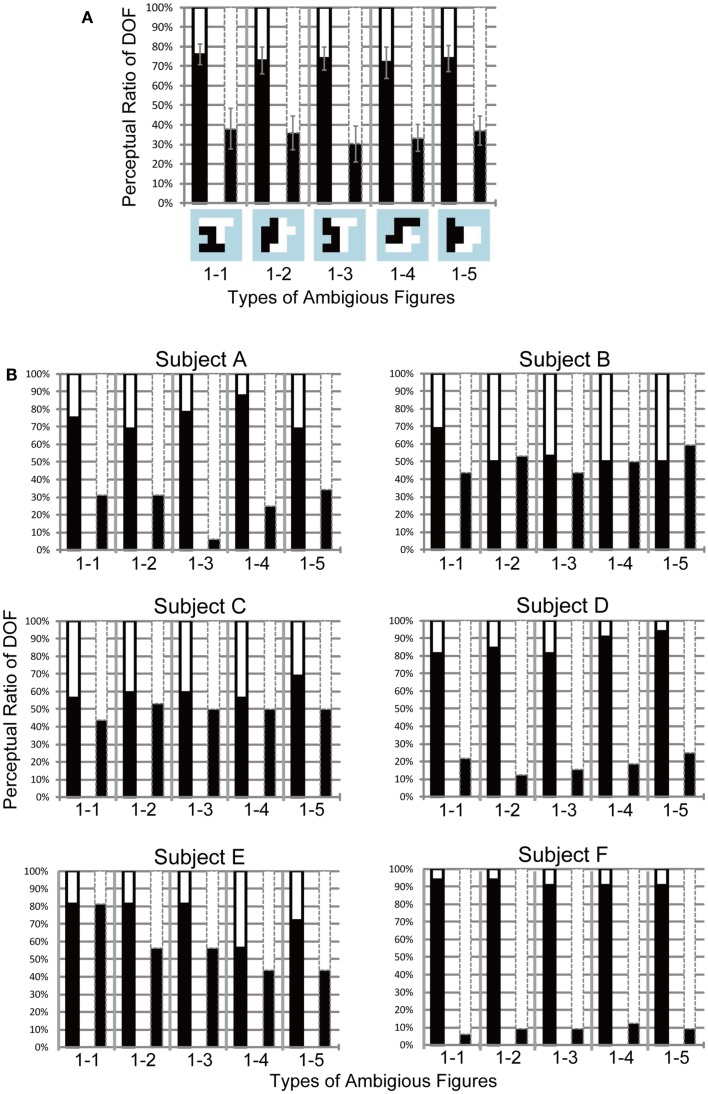
**Results of the Experiment 1 with total of six subjects (A) and each of them (B)**. Black bars show the apparent perception of DOF left for each base-type ambiguous figures indicated at the bottom of **(A)**. Two conditions for attending to the direction of motion are indicated by black solid and gray dashed contours on bar graphs, which show that black and white objects, respectively, were directed to attention. **(A)** The averaged perception of DOF from all six subjects. Error bars showed the standard error. In all types of test stimuli, significant differences were found between attention conditions (*P* < 0.01). **(B)** Results of the Experiment 1 for each subjects. The conventions were same to **(A)**. Subjects A, D, E, and F indicated the significant modulations of the DOF perception with dependence on attention conditions (A, D, F: *P* < 0.01, E: *P* < 0.05). We did not find the significant attention modulation on other subjects (B: *P* = 0.48, C: *P* = 0.12).

We investigated psychophysically the influence of feature-based attention on the determination of DOF. Participants judged DOF at the border between two adjacent regions (each consisting of an RDP moving toward a distinct direction) while directing their attention toward a particular direction of motion. All subjects reported the correct response in over 90% of the cue task. We expected that the region with the attended direction of motion would be chosen as figure more frequently. Figure 4A illustrates the shapes of the test stimuli (ambiguous figures comprising two objects) used in Experiment 1. Figure 4B shows the measured perceptual ratio of DOF, indicating the region that tended to be perceived as a figure. Note that we combined the data for each test stimulus from all mirror images and polarities of motion directions and from all participants who showed sensitivity to attention, to extract the effect of attention. To quantify the effect of feature-based attention on the perception of DOF, we carried out three-way ANOVA using two attention conditions (attend to the motion direction of left or right object), five ambiguous figures (types 1–1 through 1–5), and six participants (A, B, C, D, E, and F; five males and one female). There were significant main effects on attention (*P* < 0.001) and participants (*P* < 0.05) and significant interactions among the three factors (*P* < 0.005). The interactions led us to examine the simple main effects of each factor. Four participants (A, D, E, and F) showed significant differences in the perception of DOF with regard to two attention conditions (*P* < 0.001). The other two participants (B and C) did not exhibit significant differences (*P* = 0.48 and 0.12). We excluded the data of participants B and C from further analyses, because subsequent examinations focused on the nature of the modulation for the determination of DOF afforded by feature-based attention. The results obtained for all six participants, including individual data, are shown in Figure A1 in Appendix. Note that the tendency of the attention modulation of participants B and C was similar to other four participants although their magnitudes were small. Tzvetanov’s et al. (2006) experiment with RDP stimuli also reported the inter-subject variability while the averaged data of all subjects exhibited expected effects. The interaction between the attention condition and four participants (A, D, E, and F) was significant (two-way ANOVA, *P* < 0.001). The modulation afforded by attention was significant on all types of ambiguous figures (pairwise *t*-test, *P* < 0.005). These results indicate that the mean of all six participants exhibited a significant modulation by feature-based attention in the perception of DOF (with dependence on subject and stimulus type), and that four out of six participants exhibited a significant modulation that was independent of stimulus type (with dependence on subject). The amount of perceptual modulation by spatial attention in a similar experiment (Wagatsuma et al., 2008) was around 30–40%, whereas the amount of perceptual modulation by feature-based attention in the present experiment was around 45–65%. Although a direct comparison between the experiments is difficult, it appears that the modulation by feature-based attention is similar to (or maybe slightly larger than) the modulation by spatial attention.

### The perception of DOF and the size of the object – Experiment 2

The result of Experiment 1 indicated that feature-based attention modulates the perception of DOF, so that participants tended to judge the region with an attended motion as figure. In this section, we investigated the features that are modulated by feature-based attention in the perception of DOF. Specifically, we examined whether the mount of the attended motion direction directly modulates the perception of DOF. The test stimuli used in Experiment 1 consisted of two random-block objects with the same area, aiming at canceling the imbalance of the object size. If the motion-selective cells directly underlie the modulation of DOF, larger objects will be perceived more frequently as figures and show more attention modulation. In this section, we examined whether the modulation of DOF depends on the imbalance of the size between the objects.

**Figure 5:**
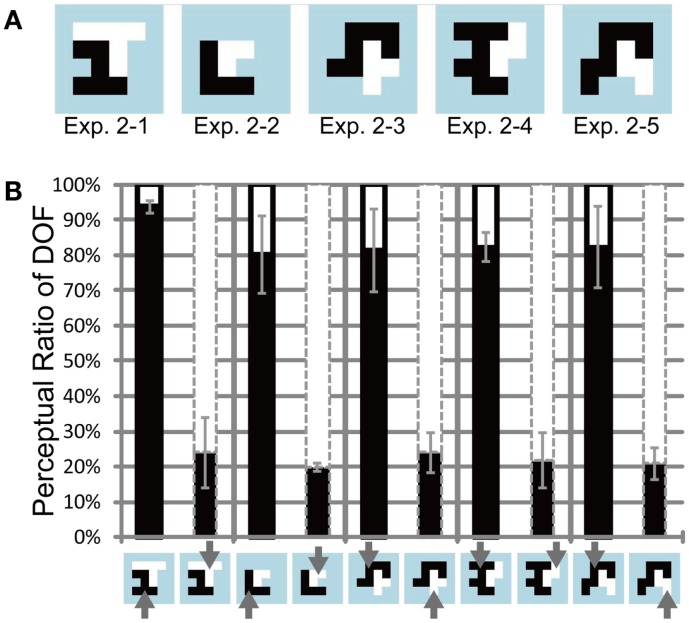
**Test stimuli and results of Experiment 2**. **(A)** Five base-type random-block stimuli. The areas of two objects (shown in black and white) were unbalanced. **(B)** Results of Experiment 2 with three participants and using the same conventions as those shown in Figure 4. The regions with attended motion direction were perceived more frequently as figure than were the alternative regions. The magnitude of modulation was independent of stimuli, indicating the irrelevance of the object size. See text for details.

**Figure A2:**
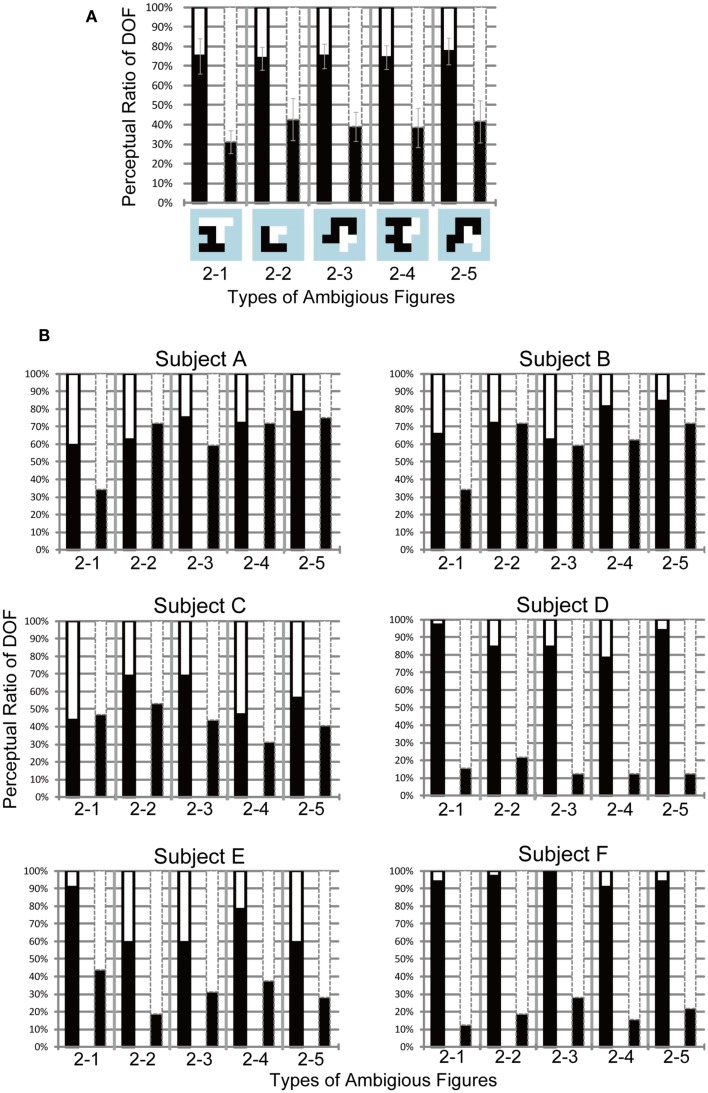
**Results of the Experiment 2 with six subjects (A) and each of them (B), with the same conventions as those for Figure A1**. **(A)** The averaged perception of DOF from all six subjects. In all test stimuli, significant differences were found between attention conditions (*P* < 0.01). **(B)** Results of the Experiment 2 for each subjects. Subjects D, E, and F indicated the significant modulations of the DOF perception with dependence on attention conditions (D, F: *P* < 0.01, E: *P* < 0.05). We did not find the significant attention modulation on other subjects (A: *P* = 0.34, B: *P* = 0.097, C: *P* = 0.086).

The test stimuli used in Experiment 2 are illustrated in Figure 5A: the ratio of the areas between the two objects ranged between 0.5 and 1.0. The shapes of the objects were identical to those used in the previous study of spatial attention (Wagatsuma et al., 2008; see Appendix). Six participants (all males) participated in the experiment. Figure 5B shows the measured perceptual ratio of DOF for all participants who indicated sensitivity to attention, using conventions that were identical to those shown in Figure 4B. Three out of six participants (A, B, and C) were excluded from further analyses and Figure 5B because they did not exhibit significant attention-based modulation (ANOVA, *P* = 0.34, 0.10, and 0.09, respectively). The results obtained for the six participants, including individual data, are presented in the Appendix (Figure A2 in Appendix). Note that the mean of all six participants exhibited a significant modulation by feature-based attention in the perception of DOF. The results suggest that the objects with attended motion are perceived more frequently as figure. To quantify the data, we performed three-way ANOVA using two attention conditions (attention to the left or right side motion direction), five types of ambiguous figures (type 2–1 through 2–5), and the three participants who exhibited significant attention modulation (D, E, and F). We observed a significant main effect of attention (*P* < 0.005), but no significant main effect on types of ambiguous figures (*P* = 0.439) and participants (*P* = 0.229). These results indicate that the significant modulation of the perception of DOF originates from feature-based attention and suggest the independence of modulation from stimulus type. Although we observed a significant interaction between participants and attention (*P* < 0.01), the tendency observed among the participants was identical (with different levels of magnitude). These results indicate that the modulation by feature-based attention is independent of the object size. To verify the modulation of the perception of DOF for each ambiguous figure and each subject, we carried out pairwise *t*-tests for each type of test stimulus and each subject. For all types of stimuli, all participants showed a significant difference in the perception of DOF between the attention conditions (all stimulus types, *P* < 0.01). This result further supports the irrelevance of the object size to attention modulation in the perception of DOF. The results of Experiment 2 indicate that the size of the object does not directly underlie the modulation of BO.

### Difference in motion direction for the attention modulation of DOF – Experiment 3

**Figure 6:**
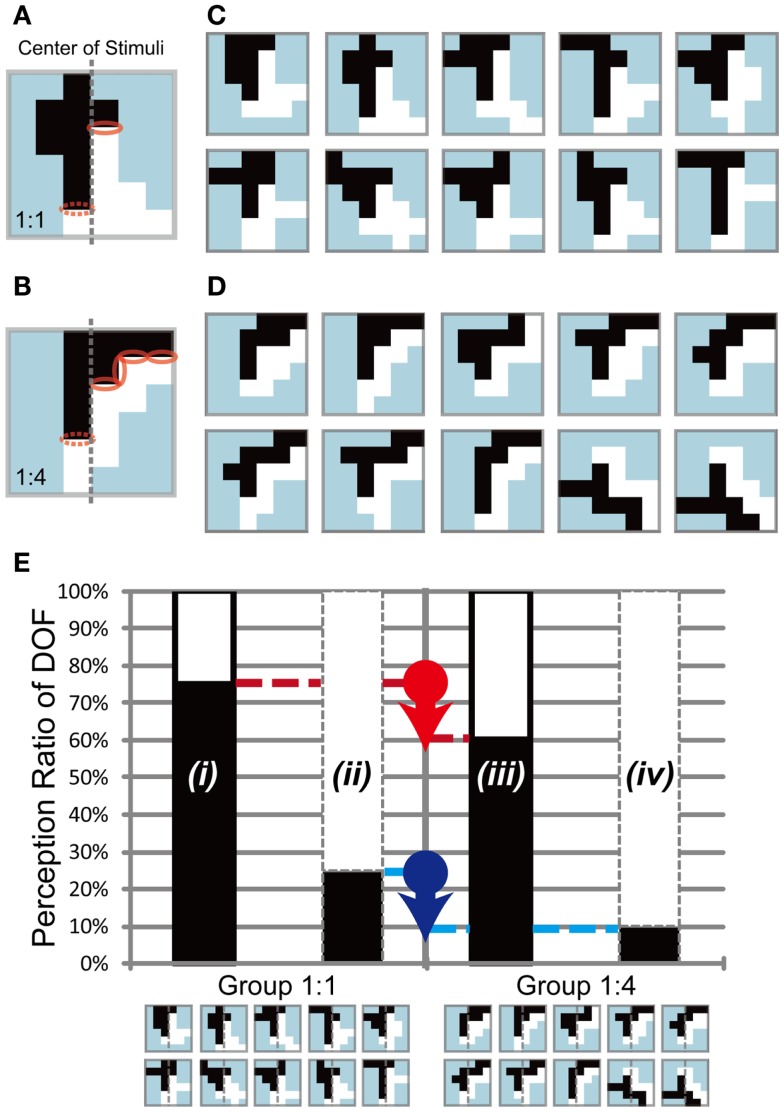
**Test stimuli and expected results of Experiment 3**. Two sets of stimuli with 1:1 **(A,C)** and 1:4 **(B,D)** motion direction differences, respectively. Examples of a balanced **(A)** and an unbalanced **(B)** stimulus. The center on the ambiguous figure was shown by the gray dashed line. Motion direction difference was equivalent to the length of border between black and white objects, which was shown by the red solid and dashed ellipses on **(A,B)**. The red dashed ellipses presented the motion direction difference on the left side. The solid ellipses indicated the right side’s motion direction difference. The motion direction difference of the stimuli in **(A,C)** was balanced, so that the border lengths of the left [dashed ellipse in **(A)**] and right [solid ellipse in **(A)**] side with respect to the center were identical, whereas those in **(B,D)** were unbalanced {the border lengths were 1 and 4 in the left [dashed ellipses in **(B)**] and right [solid ellipses in **(B)**] sides, respectively}. **(E)** Illustration of the expected results. The left and right panels show the expected results for stimuli with balanced (Group 1:1) and unbalanced (Group 1:4) motion direction difference, respectively. If the motion direction difference underlay the modulation of DOF, participants would perceive a figure more frequently in the direction with more motion direction difference. In the example of Group 1:4, the right side included more motion direction difference than the left side; thus, participants would tend to observe white objects as a figure more frequently. The red and blue arrows indicate the modulations evoked by the difference in motion direction differences, and *i* to *iv* indicate the types of modulation. See text for details.

In the previous section, we showed that the modulation of DOF by feature-based attention was independent of the object size. In this section, we examined the role of motion direction differences in the modulation afforded by feature-based attention regarding the perception of DOF. As described above, we defined *motion direction difference* as the difference in motion direction along the border between figure and ground. In our experiments, the difference in motion direction was set to 90°, thus the length of the border was proportional to the motion direction differences (Figures 6A,B). Recent accumulative data indicate that attention modulates the responses of early visual areas (Ito et al., 1998; Posner and Gilbert, 1999) and enhances feedforward processing (Wagatsuma et al., 2008; Zhang and Luck, 2009). Furthermore, computational models imply that the distribution of low-level feature contrast extracted in early vision is the basis of the determination of DOF (Sakai and Nishimura, 2006; Sakai et al., 2012). These works suggest that a factor extracted in the early vision, such as the edge or contrast of low-level features, plays an important role in attention modulation. We designed two sets of test stimuli: a *balanced* stimulus, with an equal amount of motion direction difference on the border between two objects (1:1; Figures 6A,C), and an *unbalanced* stimulus, with unequal amounts of motion direction difference on each side (1:4; Figures 6B,D). If feature-based attention had an effect on motion direction difference, the side with a higher amount of (or stronger) motion direction difference would be modulated effectively; thus, participants would tend to perceive this side as figure more frequently (Figure 6E). On the other hand, the side with a lower amount of (or weaker) motion direction difference would be modulated ineffectively; thus, participants would tend to perceive this side as a figure less frequently. The comparison between the two sets of stimuli aimed to clarify whether attention modulation of BO depends on difference in motion direction. See Appendix for the generation algorithm of balanced and unbalanced stimuli. The test stimuli used in Experiment 3 consisted of two random-block objects with the same size.

**Figure 7:**
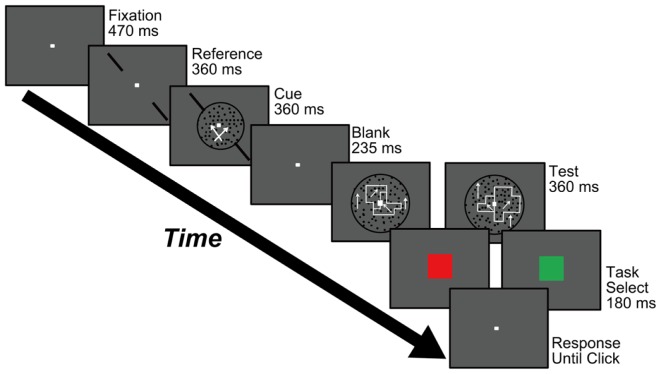
**Procedure used in Experiment 3**. Participants were asked to carry out a motion-discrimination task (stimulus shown during the “Cue” screen) and either a DOF-discrimination task or a size-discrimination task (stimulus shown during the “Test” screen). The instruction to judge DOF or size was given in the “Task Select” screen (red and green squares for DOF and size task, respectively). The participants were instructed to respond to tasks at the end of each trial, *via* 2AFC. The stimuli used in the size-discrimination task are shown in Figure A3 in Appendix.

**Figure A3:**
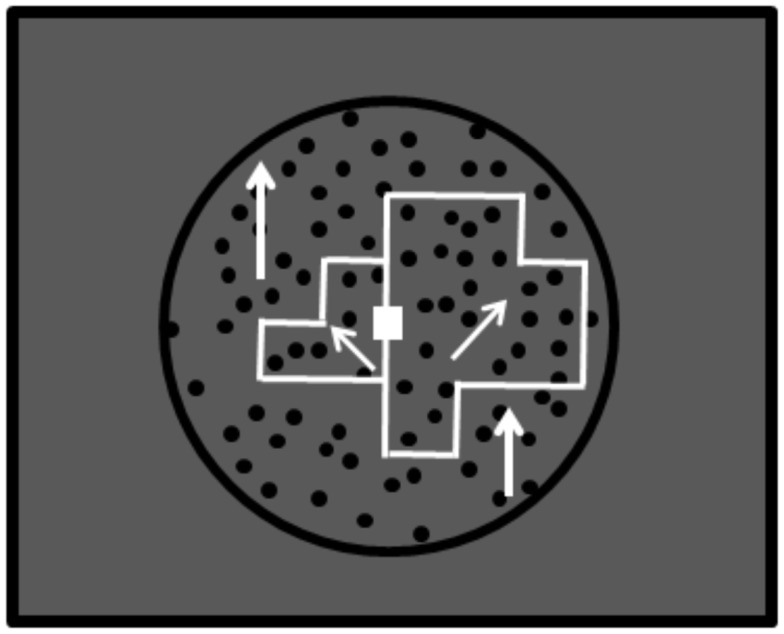
**The stimulus for the size-discrimination task of Experiment 3**. This was presented with the green square by way of compensation of the DOF-discrimination task (see Figure 7 in the main text). This task leaded subjects to see the whole of the stimulus display with more concentration. Subjects were required to answer which object was bigger through the mouse click. All subjects reported precious answers for the size-discrimination tasks over 90%.

The two sets of test stimuli used in Experiment 3 are shown in Figures 6C,D. These stimuli have more complex shapes than those used in Experiments 1 and 2, because of greater diversity in the combination of block squares. Accordingly, we modified slightly the experimental procedure to yield perceptual clarity similar to that used in the preceding experiments. The modified procedure is illustrated in Figure 7. The secondary task of size-discrimination was added to achieve a greater level of concentration by the participants (Figure A3 in Appendix). After the cue stimulus, in half of the trials participants were asked to report on the object (right or left) that was bigger. This modification led participants to concentrate on the whole stimulus display. To cancel any biases in the perception of DOF, the “Task select” screen was presented just prior to “Response” screen. One single set of the experiment consisted of 160 trials: 80 trials for DOF- and 80 trials for size-discrimination tasks. The experiment comprised eight sets (160 × 8 = 1280 trials). Five participants with normal or corrected-normal vision (two females and three males) participated in this experiment.

**Figure 8:**
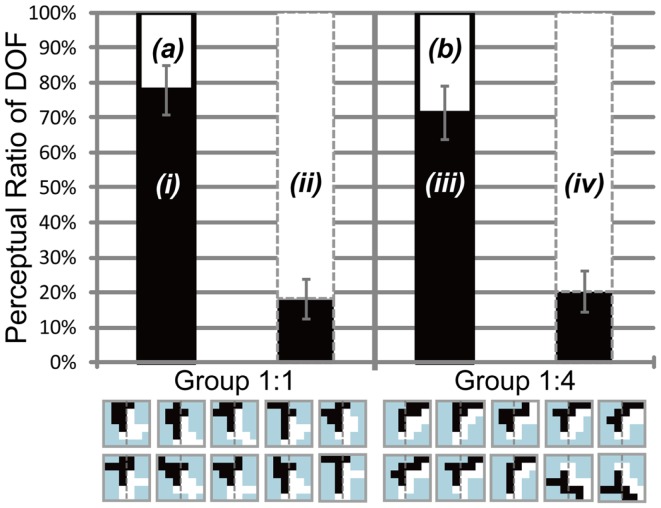
**Results of Experiment 3, which was performed using the same conventions as those shown in Figure 4**. The two bars in the left panel indicate the results obtained using balanced motion direction difference (Group 1:1) as a control. The two bars in the right panel indicate the results obtained using unbalanced (Group 1:4) motion direction difference, with a higher amount of motion direction difference in the right half of stimuli (the side of the white object) than in the alternative half (the side of the black object). Gray dashed lines on the stimulus icons mean the center of the each of them. Participants perceived figure more frequently in the direction with a higher amount of motion direction differences when attending the region with a lower amount of motion direction difference [white in the left bar surrounded by black solid contour of Group 1:4 (b) is higher than that of Group 1:1 (a)]. No such difference was observed when attending the region with a higher amount of motion direction difference [white in the bar surrounded by gray dashed contour shown by *(ii)* and *(iv)*]. See text for details.

**Figure A4:**
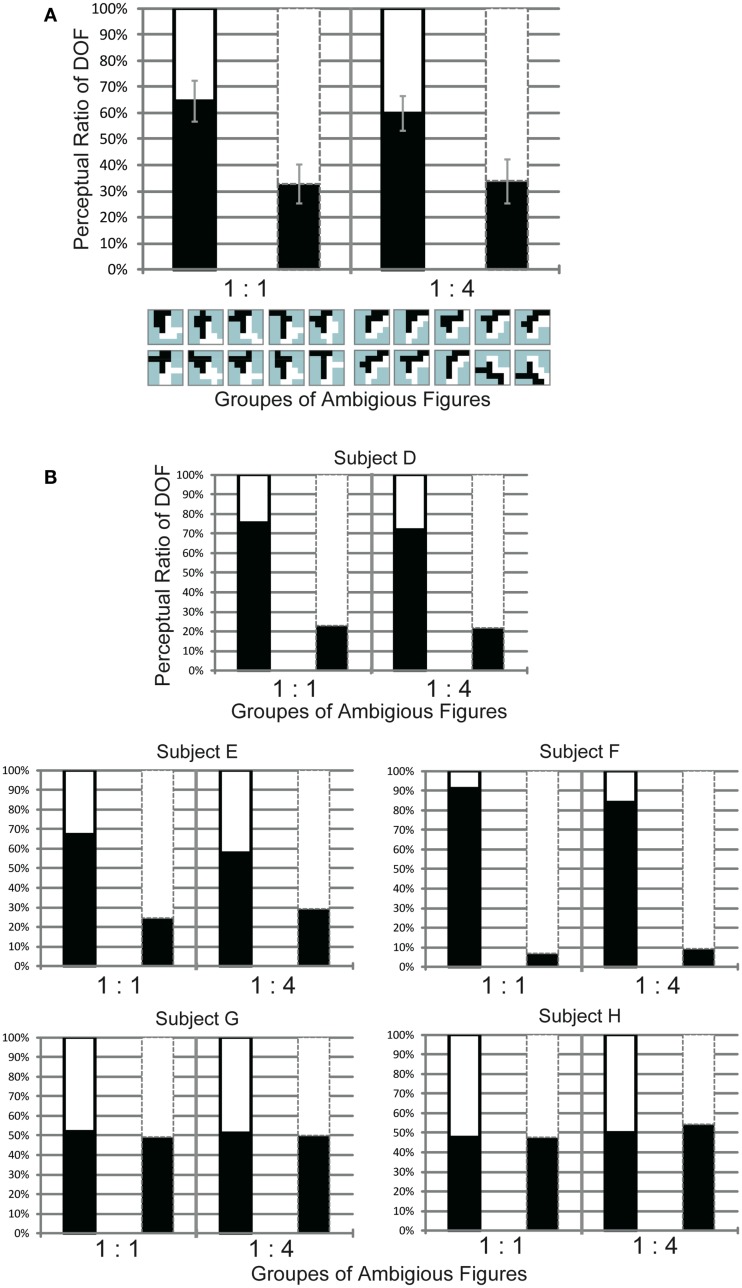
**Results of Experiment 3 from all five subjects (A) and each of them (B), with the same conventions as those for Figures A1 and A2**. The two bars in the left panel presented the results obtained using balanced edge formed by motion direction differences (Group 1:1). The two bars in the right panel were results obtained using unbalanced (Group 1:4) edge formed by motion direction differences, with more feature contrast in the right half of stimuli (the side of the white object) than in the alternative half (the side of the black object). Note that data for all stimuli of one group, mirror images, and polarities of motion directions for were combined for each bar. **(A)** The averaged perception of figure from all five subjects. There was significant modulation on the DOF perception with dependence on attention to the specific direction of motions regardless of the sets of test stimuli (*P* < 0.01). **(B)** Results of the Experiment 3 for each subjects. Subjects D, E, and F indicated the significant modulations of the DOF perception with dependence on attention conditions (D, E, F: *P* < 0.01). Other subjects did not show the significant attention modulations for DOF perception (G: *P* = 0.53, H: *P* = 0.49).

We carried out this experiment to investigate the role of motion direction difference in attention modulation. If difference in motion direction underlies this modulation, as illustrated in Figure 6E, it is expected that the side with a higher amount of motion direction difference evokes more modulation and tends to be perceived as figure more frequently. Figure 8 shows the results obtained for three participants (D, E, and F). Because this experiment focused on the cause of attention modulation, we excluded two of the five participants, as they did not show significant modulation by attention (ANOVA, *P* = 0.52 and 0.49, respectively). The results obtained for all five participants, including individual data, are given in the Appendix (Figure A4 in Appendix). Note that the mean of all five participants exhibited a significant modulation by feature-based attention in the perception of DOF. To analyze whether difference in motion direction is essential for attention modulation, we carried out three-way ANOVA using the factors of attention (attention to the left or right), motion direction difference (balanced and unbalanced), and participants (D, E, and F). We observed a significant main effect of attention (*P* < 0.001), indicating the modulation by attention for these stimuli. We also observed the presence of significant interactions between attention and participants (*P* < 0.001) and between attention and motion direction difference (*P* < 0.001). The interaction between attention and subject led us to examine the simple main effects of attention and subject. This analysis indicated the presence of significant differences in the magnitude of attention modulation among participants, with all participants showing significance in attention modulation (*P* < 0.001). Similarly, we examined the interaction between attention and motion direction difference. The analysis of simple main effects showed that motion direction difference was a significant factor when attending to the side with a lower amount of motion direction difference, but not to the side with a higher amount of motion direction difference. This implies, based on the illustration presented in Figure 6E, that the red arrow was significant, whereas the blue arrow was not. This result indicates that the effect of attention is significantly smaller in the direction of a lower amount of motion direction difference (iii in Figure 8) compared with the effects of stimuli with balanced motion direction difference (i = ii). On the other hand, the magnitude of attention modulation was indistinguishable between the directions of a higher amount of motion direction difference and balanced stimuli (*P* = 0.086). These analyses show that a higher amount of motion direction difference (iv) is effective in attention modulation, whereas a lower amount of motion direction difference (iii) is significantly less effective. The cause of this asymmetry is discussed below (see Discussion). The results of Experiment 3 indicate that the feature contrast arising from the differences between two directions of motion plays a crucial role in the modulation of DOF by feature-based attention. These results do not conflict with the computational model that BO signaling is determined based on the balance of surround low-level feature contrast between both sides of its CRF, not the feature itself (Sakai and Nishimura, 2006).

### Neural mechanisms underlying the modulation of BO by feature-based attention – the abstract V1–V2 network model

The results described in the previous section showed the crucial role of motion direction difference in the modulation of BO. This result led us to hypothesize that feature-based attention acts on low-level feature contrast extracted in early vision; thus, the modulated contrast changes the activities of BO-selective neurons in V2. Given that BO-selective cells signal the side of BO based on surround contrast (Sakai and Nishimura, 2006; Sakai et al., 2012), the modified contrast would modify directly the response of the cells. If this modification was strong, the side of BO could even be flipped, as observed for ambiguous figures. Essentially, this mechanism shares the framework of spatial attention for the determination of BO (Wagatsuma et al., 2008). Furthermore, if attention modulation of low-level feature contrast played a key role for the modulation of the responses of BO-selective cells and the switching of the perception of figure, it is expected that the magnitude of the perceptual modulation on the DOF is invariant among types of stimuli since attention in early vision might change low-level feature contrast prior to the binding to or perception of stimulus shapes. To investigate the plausibility of the mechanism of feature-based attention, we constructed an abstract V1–V2 network model (as shown in Figure 3A) and analyzed its behavior. In this model, feature-based attention presumably originates in higher visual areas such as MT. The top-down feature-based attention act on V1 cells, to increase edge or feature contrast, specifically the differences in motion direction. The increased feature contrast in V1 is fed to BO-selective cells in V2 and modifies their activities according to the modulation of contrast (Figure 3B). In short, dots with attended direction of motion have increased motion direction difference, and such “easy-to-see” dots tend to attract the direction of BO toward them. A detailed description of the model is given in the Section “Materials and Methods.” Parameters of this model were fixed through all simulations. The modulation of the responses of our model with a type of stimulus emerged from the attention conditions. We examined whether the model BO-selective cells reproduced the results of our psychophysical experiments, which were described in the preceding sections.

#### Feature-based attention in early vision for the modulation of BO-selective neurons

**Figure 9:**
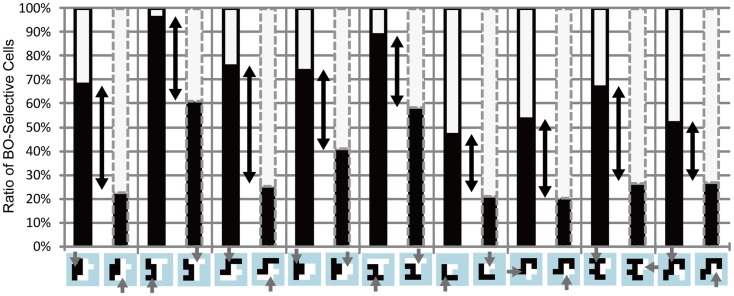
**Simulation results for the stimuli used in Experiments 1 and 2**. Black bars indicate the ratio of the activities of BO-left model cells compared with BO-right model cells. The black solid and gray dashed contours around the bars indicate that black and white objects, respectively, were attended. Black arrows represent the magnitude of attention modulation. There was no significant difference in the magnitude of attention modulation among the stimuli, which was in agreement with human perception.

The results of the psychophysical experiments showed the significant modulation of DOF by feature-based attention. To test whether the model BO-selective cells behave in a manner that is similar to perception, we performed simulations of the model with the edge maps mimicking the shapes of test stimuli used in Experiments 1 and 2 and compared the magnitude of attention modulation observed in the model BO-selective cells with that obtained for human responses. The activities of the model BO-selective cells in response to individual stimuli are shown in Figure 9. The magnitude of attention modulation is defined as the difference in the proportion of the BO-left model cells between two attention conditions, which is presented by black arrows in Figure 9. We observed attention modulation that was similar to the human responses, as shown in Figures 4B and 5B and Figures A1 and A2 in Appendix. Note that there was no direct feature-based attention effect for the BO determination in V2 stage. These attention modulations of model BO-selective cells solely emerged from a change in the activities of model cells in V1 stage. There was no significant difference in the magnitudes of attention modulation between the model and human responses of six participants average (ANOVA, *P* = 0.500), suggesting the plausibility of the model as representative of the modulation of BO by feature-based attention. According to the model, no significant difference was observed in the magnitude of the attention modulation among stimuli (ANOVA, *P* = 0.137), indicating the shape invariance of the modulation. To quantify the shape variance in the psychophysical experiments, we carried out two-way ANOVA of the results of Experiments 1 and 2 using factors of stimulus type (*n* = 9) and participants (*n* = 6). The results of this analysis did not show significance for the main factors, indicating the invariance of shape and subject in attention modulation. The shape invariance shown for both simulations and human perceptions supports the hypothesis that feature contrast modulation in early vision is crucial for the modulation of the activities of BO-selective neurons. Shape invariance has also been reported in spatial attention (Wagatsuma et al., 2008). The agreement between the model and psychophysical results suggests that the modulation of low-level feature contrast in early vision underlies the modulation of BO-selective neurons in V2.

#### Feature contrast dependence in the modulation of BO

**Figure 10:**
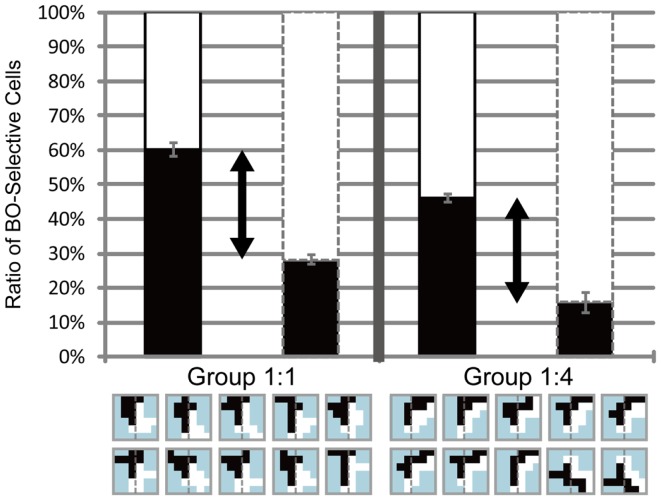
**Simulation results for the stimuli used in Experiment 3**. The left and right panels show the simulation results for stimuli with balanced (Group 1:1) and unbalanced (Group 1:4) edge or contrast of feature. Black arrows represent the total of the magnitude of attention modulation. The model reproduced the characteristics observed in Experiment 3, with the exception of the asymmetry in attended objects regarding strong and weak contrast. See text for details.

Experiment 3 showed the presence of motion direction difference dependence in the modulation of DOF by feature-based attention. We performed simulations of the model with edge maps mimicking the shapes of test stimuli used in Experiment 3, to investigate whether the activities of model BO-selective cells depend on the amount of edge formed by motion direction difference. Figure 10 shows the computed responses of model BO-selective cells, as categorized by difference in motion direction (Group 1:1 and 1:4). We observed attention-based modulation in the determination of BO. Two-way ANOVA using the factors of attention and motion direction difference (with the repetition of individual stimuli) was significant for the main factors (*P* < 0.001), but not for the interaction (*P* = 0.67), indicating the dependence of the model cell activities on attention and motion direction difference. These results suggest that the model determines BO on a specific side if that side includes a large amount of edge formed by motion direction difference compared with the other side, and if the side is attended.

Experiment 3 showed the asymmetry in the attention modulation with respect to the amount of motion direction difference: the modulation was less effective if attended to the side with a small amount of motion direction difference, but was similarly effective if attended to a large amount of motion direction difference compared with the equal motion direction difference in balanced stimuli. The model did not show this asymmetry. The analysis of the simple main effect of attention showed the significance independent of edge formed by motion direction difference (ANOVA, *P* = 0.66). This result indicates that the amount of the modulation is similarly independent of the strength of edge formed by motion direction difference to which attention is directed. After the description of Experiment 3, we speculated that a large amount of edge based on motion direction difference did not alter the modulation because the regular edge formed by motion direction difference in balanced stimuli reaches the level at which the modulation is saturated. As our model did not include the compressive non-linearity that establishes saturation, a large amount of edge formed by motion direction difference yielded enhancement in modulation. The issue of this asymmetry is discussed below (see Discussion). The simulations of the model with the stimuli used in the psychophysical experiments supported the hypothesis that the mechanism based on the modulation of low-level feature contrast by attention, presumably in early vision, plays a crucial role in the modulation of DOF by feature-based attention, similarly to spatial attention.

## Discussion

We investigated whether and how feature-based attention modulates F–G segregation. Using ambiguous figures consisting of a combination of RDPs moving toward orthogonal directions, we conducted psychophysical experiments to examine whether the perception of BO is modified by directing feature-based attention to the direction of motion. Our results showed that feature-based attention alters the perception with a degree that is comparable to that of spatial attention. Furthermore, the modulation of perception was independent of stimulus types. These results support a crucial contribution of the early visual areas to the modulation of attention. Subsequent experiments showed that F–G perception is dependent on the distribution of difference in motion direction, but independent of the object size. Based on these results, we constructed a computational model for modulation by feature-based attention. In the model, top-down feature-based attention modulates the low-level feature contrast, specifically edge formed by motion direction difference, in V1, and this modulation of the feature contrast changes directly the surround contrast of BO-selective cells in V2. The simulations of the model showed good agreement with human perception regarding the magnitude of attention modulation and its invariance among stimuli. These results indicate that the contrast of early level features that are modified by feature-based attention alter subsequent processing along afferent pathways, and that such modification could even change the selection of an object during F–G segregation. This mechanism is essentially identical to that observed in spatial attention, with the exception that spatial attention modulates cells that share a retinotopic location, whereas feature-based attention modulates cells that share a feature.

The results of our psychophysical experiments indicated that the magnitude of feature-based attention for DOF perception is independent among the types of stimuli. This shape invariance suggests the influences of attention in early visual areas for the modulation of DOF perception (Wagatsuma et al., 2008). The attention effects of the proposed network model for BO-selective cells were limited to the V1 stage. However, this model not only showed the modulation of the activities of BO-selective cells and the flip of DOF perception, but also reproduced the shape invariance on the magnitude of attention modulation. The agreement between the psychophysics and the corresponding simulations supports the validity of the proposed model and suggests that attention modulation of low-level feature contrast in early visual area underlies the flip of BO determination. These results predict that attention modulation of DOF perception originates, at least in part, from modulation of low-level feature contrast in early vision sensitivity.

Our main result was that motion-based attention modulates the perception of DOF. Participants directed their attention toward a specific direction of motion through the motion-discrimination task utilized by Tzvetanov et al. (2006). Their study indicated the validity of this task for regulating participants’ motion-based attention. Followed by this cue stimulus, we presented the blank screen for 235 ms until the presentation of test stimulus (Figure 2). The presentation of such interval (blank screen) is crucial to assure the effect of feature-based attention. A physiological study reported that the effects of both spatial and feature-based attention were apparent with a randomized interval of 200–400 ms (Cohen and Maunsell, 2011). The recent psychophysical work reported that motion-based attention took 300–500 ms to exert its effect (Liu et al., 2007b). Other studies often used 200–3000 ms of ISI or SOA for studying feature-based attention (Lu and Itti, 2005; Katzner et al., 2009). These works suggest the behavioral latency of feature-based attention, supporting that motion-based attention underlies our observation of the modulation of DOF.

It is the main purpose of this work to understand how feature-based attention modulates the F–G segregation, so that we statistically analyzed the data of participants with sensitivity to attention. Some of participants did not exhibit significant effects of attention. Other psychophysical works also reported the inter-subject variability (Grunewald, 2004; Tzvetanov et al., 2006). However, the average of all participants showed significant attention modulation on DOF perception (Figures A1, A2, and A4 in Appendix), which suggested the validity of feature-based attention. We found that stable effects of attention were observed from the expert or well trained subjects (D, E, and F). The possible explanations for this difference among participants are the difficulty of the experiments and the training of participants. The procedure of the present experiments is similar to our previous experiments on spatial attention (Wagatsuma et al., 2008). In these previous experiments, a flashing dot directed the attention toward a specific location, without an additional task or a response to this cue stimulus. However, the present experiments consisted of dual tasks, motion- and DOF-discrimination tasks. The increase of the complexity and difficulty of the experiments might decrease the performance of naïve subjects.

The result of Experiment 2 showed the irrelevance of object size to attention modulation in the perception of DOF. This result led to the hypothesis that the modulation by feature-based attention is independent of motion energy that is considered a crucial, low-level feature processed in early visual areas (Adelson and Bergen, 1985; Landy and Bergen, 1991; DeAngelis et al., 1993). The amount of motion energy is proportional to the amount of area, because larger areas include a greater number of moving RDPs. However, the result of Experiment 2 indicated the independence of object size for the feature-based attention modulation, suggesting that motion energy does not underlie the modulation by feature-based attention. Although this prediction appears to be key for understanding the mechanism of visual attention, our present experimental methods appear to be not appropriate to discuss attention effects for the motion energy. A further study is necessary to understand the attention modulation of the motion energy.

Our Experiment 3 indicated that the modulation of DOF by attention was dependent on the distribution of differences in motion direction and that the modulation was asymmetric between *balanced* and *unbalanced* stimuli (Figure 8). Specifically, a large amount of motion direction differences was effective in attention modulation, whereas a small amount of motion direction difference was significantly less effective. It was not clarified whether this asymmetry is essential for attention modulation. The possible explanations for this asymmetry include the saturation of modulation. The modulation to the direction of a large amount of motion direction differences reached 80%; thus, further modulation may be difficult to measure. It could also be considered that a small amount of motion direction differences is less effective for the determination of DOF rather than the attention modulation. This alternative explanation could be examined by measuring the ratio of DOF perception for the present stimulus sets without directing attention. However, this measurement is sensitive to other cues, such as the shape and size of block stimuli, and would not help support this explanation. Our model did not reproduce this asymmetry, possibly because the model did not include either the mechanism of the saturation or the dependence of DOF on low-level feature contrast. The investigation on the quantitative behavior of the modulation may lead to further understanding of the mechanisms underlying the selection of surface by feature-based attention.

## Conflict of Interest Statement

The authors declare that the research was conducted in the absence of any commercial or financial relationships that could be construed as a potential conflict of interest.

## Appendix

### Generations of pseudo random-block objects and ambiguous figures

We carried out psychophysical experiments with pseudo white-noise random-block objects to investigate whether feature-based attention modulates the perception of F–G segregation. The pseudo white-noise random-block objects approximate all possible shapes under certain conditions. The method for the generation of pseudo white-noise random-block objects is given here.

A block object consisted of *n* square blocks placed on *m* × *m* grids, with center 2 × 2 grids were fixed with the two blocks placed on one side of the center so that contrast within the CRF was kept identical for all stimuli. The other blocks (*n*−2) were placed randomly adjacent to the existing blocks except for the CRF region. We call this set of stimuli as *n-block stimuli*. Stimulus shape is more complex with a larger number of blocks and grids. In this paper, the number of grid for Experiment 1 was fixed to 4 × 4 and that of blocks were 6. That of grid for Experiment 2 was 4 × 4 and that of blocks were 3, 4, 6, and 8. Stimulus shape used in Experiment 3 is more complex. The number of grid was 6 × 6 and that of blocks were 8.

We brought together two block stimuli to produce a stimulus with ambiguous BO at the center as illustrated in Figures 4A, 5A, and 6C,D. Ambiguous random-block stimuli were comprised of a combination of the block stimulus with BO left and that with right. All combinations of these stimuli were prepared, and if there was an overlap of blocks, we excluded such combination. For Experiment 1 and 2, because this method generated a number of stimuli, we selected five stimuli that appears most ambiguous from visual inspection of several people who did not participate in the experiments. For Experiment 3, we randomly selected 10 *balanced* (Group 1:1) and *unbalanced* (Group 1:4) stimuli from all generated combinations.

## References

[B1] Adelson E. H. Bergen J. R. (1985). Spatiotemporal energy models for the perception of motion. J. Opt. Soc. Am. A 2, 284–299 10.1364/JOSAA.2.000284 3973762

[B2] Baluch F. Itti L. (2011). Mechanism of top-down attention. Trends Neurosci. 34, 210–224 10.1016/j.tins.2011.02.003 21439656

[B3] Carrasco M. (2011). Visual attention: the past 25 years. Vision Res. 51, 1484–1525 10.1016/j.visres.2011.04.012 21549742 PMC3390154

[B4] Carrasco M. Ling S. Read S. (2004). Attention alters appearance. Nat. Neurosci. 7, 308–313 10.1038/nn1194 14966522 PMC3882082

[B5] Cohen M. R. Maunsell J. H. (2011). Using neuronal populations to study the mechanisms underlying spatial and feature attention. Neuron 70, 1192–1204 10.1016/j.neuron.2011.04.029 21689604 PMC3579499

[B6] Craft E. Schütze H. Niebur E. von der Heydt R. (2007). A neural model of figure-ground organization. J. Neurophysiol. 97, 4310–4326 10.1152/jn.00203.2007 17442769

[B7] DeAngelis G. C. Ohzawa I. Freeman R. D. (1993). Spatiotemporal organization of simple-cell receptive fields in the cat’s striate cortex. II. Linearity of temporal and spatial summation. J. Neurophysiol. 69, 1091–1117 8492152 10.1152/jn.1993.69.4.1118

[B8] Deco G. Lee T. S. (2004). The role of early visual cortex in visual integration: a neural model of recurrent interaction. Eur. J. Neurosci. 20, 1089–1100 10.1111/j.1460-9568.2004.03528.x 15305878

[B9] Gregoriou G. G. Gotts S. J. Zhou H. Desimone R. (2009). High-frequency, long-range coupling between prefrontal and visual cortex during attention. Science 324, 1207–1210 10.1126/science.1171402 19478185 PMC2849291

[B10] Grunewald A. (2004). Motion repulsion is monocular. Vision Res. 44, 959–962 10.1016/j.visres.2003.10.027 15031088

[B11] Hasson U. Hendler T. Bashat D. B. Malach R. (2001). Vase or face? A neural correlate of shape-selective grouping processes in the human brain. J. Cogn. Neurosci. 13, 744–753 10.1162/08989290152541412 11564319

[B12] Ito M. Westheimer B. Gilbert C. D. (1998). Attention and perceptual learning modulate contextual influences on visual perception. Neuron 20, 1191–1197 10.1016/S0896-6273(00)80499-7 9655506

[B13] Itti L. Koch C. (2001). Computational modeling of visual attention. Nat. Rev. Neurosci. 2, 194–203 10.1038/35058500 11256080

[B14] Jones H. E. Grieve K. L. Wang W. Sillito A. M. (2001). Surround suppression in primate V1. J. Neurophysiol. 86, 2011–2028 11600658 10.1152/jn.2001.86.4.2011

[B15] Jones H. E. Wang W. Sillito A. M. (2002). Spatial organization and magnitude of orientation contrast interactions in primate V1. J. Neurophysiol. 88, 2796–2808 10.1152/jn.00403.2001 12424313

[B16] Katzner S. Busse L. Treue S. (2009). Attention to the color of a moving stimulus modulates motion-singnal processing in mcaque area MT: evidence for a unified attentional system. Front. Syst. Neurosci. 3:12 10.3389/neuro.06.012.2009 19893762 PMC2773174

[B17] Landy M. S. Bergen R. (1991). Texture segregation and orientation gradient. Vision Res. 31, 679–691 10.1016/0042-6989(91)90153-V 1843770

[B18] Lee K. Itti L. Koch C. Braun J. D. (1999). Attention activates winner-take-all competition among visual filters. Nat. Neurosci. 2, 375–381 10.1038/7286 10204546

[B19] Ling S. Liu T. Carrasco M. (2009). How spatial and feature-based attention affect the gain and tuning of population responses. Vision Res. 49, 1194–1204 10.1016/j.visres.2008.05.025 18590754 PMC2696585

[B20] Liu T. Larsson J. Carrasco M. (2007a). Feature-based attention modulates orientation-selective responses in human visual cortex. Neuron 55, 313–323 10.1016/j.neuron.2007.06.030 17640531 PMC2789471

[B21] Liu T. Stevens S. T. Carrasco M. (2007b). Comparing the time course and efficacy of spatial and feature-based attention. Vision Res. 47, 108–113 10.1016/j.visres.2007.04.023 17087987

[B22] Lu J. Itti L. (2005). Perceptual consequences of feature-based attention. J. Vis. 5, 622–631 10.1167/5.8.1034 16231997

[B23] Martinez-Trujillo J. C. Treue S. (2004). Feature-based attention increases the selectivity of population responses in primary visual cortex. Curr. Biol. 14, 744–751 10.1016/j.cub.2004.04.028 15120065

[B24] McAdams C. J. Maunsell J. H. (1999). Effects of attention on the reliability of individual neurons in monkey visual cortex. Neuron 23, 765–773 10.1016/S0896-6273(01)80034-9 10482242

[B25] Mihalas S. Dong Y. von der Heydt R. Niebur E. (2011). Mechanism of perceptual organization provide auto-zoom and auto-localization for attention to objects. Proc. Natl. Acad. Sci. U.S.A. 108, 7583–7588 10.1073/pnas.1014655108 21502489 PMC3088583

[B26] Mitchell J. F. Stoner G. R. Reynolds J. H. (2004). Object-based attention determines dominance in binocular rivalry. Nature 429, 410–413 10.1038/nature02584 15164062

[B27] Ozeki H. Finn I. M. Schaffer E. S. Miller K. D. Ferster D. (2009). Inhibitory stabilization of the cortical network underlies visual surround suppression. Neuron 62, 578–592 10.1016/j.neuron.2009.03.028 19477158 PMC2691725

[B28] Palmer J. Ames C. T. Lindsey D. T. (1993). Measuring the effect of attention on simple visual search. J. Exp. Psychol. Hum. Percept. Perform. 19, 108–130 10.1037/0096-1523.19.1.108 8440980

[B29] Paradiso M. A. (2002). Perceptual and neuronal correspondence in primary visual cortex. Curr. Opin. Neurobiol. 12, 155–161 10.1016/S0959-4388(02)00311-2 12015231

[B30] Peters R. J. Iyer A. Itti L. Koch C. (2005). Components of bottom-up Gaza allocation in natural images. Vision Res. 45, 2397–2416 10.1016/j.visres.2005.03.019 15935435

[B31] Posner M. I. (1980). Orienting of attention. Q. J. Exp. Psychol. 32, 3–25 10.1080/00335558008248231 7367577

[B32] Posner M. I. Gilbert C. D. (1999). Attention and primary visual cortex. Proc. Natl. Acad. Sci. U.S.A. 96, 2585–2587 10.1073/pnas.96.6.2585 10077552 PMC33534

[B33] Qiu T. Sugihara T. von der Heydt R. (2007). Figure-ground mechanisms provide structure for selective attention. Nat. Neurosci. 10, 1492–1499 10.1038/nn1853 17922006 PMC2666969

[B34] Reynolds J. H. Heeger D. J. (2009). The normalization model of attention. Neuron 61, 168–185 10.1016/j.neuron.2009.01.002 19186161 PMC2752446

[B35] Sajda P. Finkel L. H. (1995). Intermediate-level vision representations and the construction of surface perception. J. Cogn. Neurosci. 7, 267–291 10.1162/jocn.1995.7.2.267 23961828

[B36] Sakai K. Nishimura H. (2006). Surrounding suppression and facilitation in the determination of border ownership. J. Cogn. Neurosci. 18, 562–579 10.1162/jocn.2006.18.4.562 16768360

[B37] Sakai K. Nishimura H. Shimizu R. Kondo K. (2012). Consistent and robust determination of border ownership based on asymmetric surrounding contrast. Neural Netw. 33, 257–274 10.1016/j.neunet.2012.05.006 22732320

[B38] Solmon J. A. Lavie N. Morgan M. J. (1997). Contrast discrimination function: spatial cueing effects. J. Opt. Soc. Am. 14, 2443–2448 10.1364/JOSAA.14.002443 9291612

[B39] Sugihara T. Tsuji Y. Sakai K. (2007). Border-ownership-dependent tilt after effect in incomplete figures. J. Opt. Soc. Am. A Opt. Image Sci. Vis. 24, 18–24 10.1364/JOSAA.24.000018 17164839

[B40] Treue S. Maunsell J. H. (1999). Effects of attention on the processing of motion in macaque middle temportal and medial superior temporal visual cortical areas. J. Neurosci. 19, 7591–7602 10460265 10.1523/JNEUROSCI.19-17-07591.1999 PMC6782504

[B41] Tzvetanov T. Wolmelsdorf T. Niebergall R. Treue S. (2006). Feature-based attention influences contextual interactions during motion repulsion. Vision Res. 46, 3651–3658 10.1016/j.visres.2006.05.018 16828839

[B42] Wagatsuma N. Potjans T. C. Diesmann M. Fukai T. (2011). Layer-dependent attentional processing by top-down signals in a visual cortical microcircuit model. Front. Comput. Neurosci. 5:31 10.3389/fncom.2011.00031 21779240 PMC3134838

[B43] Wagatsuma N. Potjans T. C. Diesmann M. Sakai K. Fukai T. (2012). Space-based and feature-based attention in a realistic layered-microcircuit model of visual cortex. J. Vis. 12, 662 10.1167/12.9.662

[B44] Wagatsuma N. Shimizu R. Sakai K. (2008). Spatial attention in early vision for the perception of border ownership. J. Vis. 8, 22 10.1167/8.3.22 19146255

[B45] Zhang W. Luck S. J. (2009). Feature-based attention modulates feedforward visual processing. Nat. Neurosci. 12, 24–25 10.1038/nn.2223 19029890

[B46] Zhou H. Friedman H. S. von der Heydt R. (2000). Coding of border ownership in monkey visual cortex. J. Neurosci. 20, 6594–6611 10964965 10.1523/JNEUROSCI.20-17-06594.2000 PMC4784717

